# Neutrophils Affect IL-33 Processing in Response to the Respiratory Allergen *Alternaria alternata*

**DOI:** 10.3389/fimmu.2021.677848

**Published:** 2021-08-17

**Authors:** Sharon Van Nevel, Judith van Ovost, Gabriele Holtappels, Natalie De Ruyck, Nan Zhang, Harald Braun, Tania Maes, Claus Bachert, Olga Krysko

**Affiliations:** ^1^Upper Airways Research Laboratory, Department of Head and Skin, Ghent University, Ghent, Belgium; ^2^Unit for Structural Biology, VIB-UGent Center for Inflammation Research, Ghent, Belgium; ^3^Unit for Structural Biology, Department of Biochemistry and Microbiology, Ghent University, Ghent, Belgium; ^4^Department of Respiratory Medicine, Department of Internal Medicine, Ghent University, Ghent, Belgium; ^5^Department of Ear, Nose and Throat Diseases, Karolinska University Hospital, Stockholm, Sweden

**Keywords:** allergy, protease, type 2 inflammation, elastase, asthma

## Abstract

Future precision medicine requires further clarifying the mechanisms of inflammation in the severe endotypes of chronic airway diseases such as asthma and chronic rhinosinusitis (CRS). The presence of neutrophils in the airways is often associated with severe airway inflammation, while their precise contribution to the severe inflammation is largely unknown. We aimed to study the role of neutrophils in BALB/c and C57BL/6 mice exposed to *Alternaria alternata* (*Alt*). The mice were exposed to *Alt* extract for twelve hours or ten days to induce allergic airway inflammation. C57BL/6 mice exposed to *Alt* responded with eosinophilic infiltration and the characteristic IL-5 upregulation. In contrast, the inflammatory response to *Alt* extract in BALB/c mice was characterized by a neutrophilic response, high levels of G-CSF, and elastase in the lungs. The lack of neutrophils affected the processing of IL-33 in BALB/c mice, as was demonstrated by depletion of neutrophils through intraperitoneal injections of anti-Ly6G antibody. Our data identifies the key role of neutrophils in airway inflammation through IL-33 cleavage in the *Alt*-induced airway inflammation in mice, which could potentially underline the different endotypes in human disease.

## Introduction

Severe asthma and chronic rhinosinusitis (CRS) with nasal polyps remain the most severe and often uncontrolled extremes of type 2 inflammatory diseases of the upper and lower airways (1, 2). Type 2 inflammatory responses are characterized by the production of cytokines, such as IL-4, IL-5, and IL-13, secreted by classic Th2 cells; and by innate immune cells, such as group 2 innate lymphoid cells (ILC2s), basophils, eosinophils, and mast cells. IL-33, an IL-1 family cytokine and typically linked with type 2 inflammation was associated with reduced sensitivity to corticosteroid therapy in asthmatic children and the animal models of asthma (3–5). In severe asthmatics, the levels of IL-33 are increased in bronchoalveolar lavage fluid and lung biopsies and correlate negatively with lung function (5). IL-33 is released by airway epithelial cells during their activation or passively released during cellular damage (6, 7). The IL-33 receptor (ST2, IL1R1) is expressed by a wide variety of immune cells, including mast cells, eosinophils, basophils, Th2 cells, ILC2s, and epithelial cells, and is becoming a valuable target for the treatment of IL-33 driven diseases (8). Genetic and environmental factors are being extensively studied to estimate their role in the pathophysiology and development of disease endotypes in asthma and CRS (9). Four independent cohorts [Lifelines, Dutch Asthma GWAS (DAG), Genetics of Asthma Severity and Phenotypes (GASP), Manchester Asthma and Allergy Study (MAAS)] and resequencing data have shown that IL-33 genetic signals potentially contribute to severe phenotypes in asthma (10). Severe and corticosteroid-resistant endotypes of asthma are often characterized by high neutrophil counts in sputum, increased levels of IL-33, the formation of neutrophilic traps and activation of the inflammasome (11–15). A recent study has shown that children with severe refractory asthma demonstrate neutrophilia in the bronchiolar lavage fluid (BALF) and a strong Th17 and Th1 cytokine response (16). Also, a longitudinal study over three years in asthmatic patients showed that the baseline sputum inflammatory phenotype with high eosinophils and neutrophils numbers didn’t change over time and could predict the reduction in lung function (17). Upon allergen challenge, IL-33 is released as a full-length form with limited biological activity, further processed by secreted endogenous proteases. The proteases regulating the activity of IL-33 may be released from activated mast cells (chymase, tryptase, and granzyme B) and neutrophils (cathepsin G, elastase, and proteinase 3) (18). The proteolytic cleavage of IL-33 in its activation domain results in the generation of shorter “mature” forms of IL-33 that are at least 10-30 fold more potent than full-length IL-33 in the induction of type 2 inflammation and activation of ILC2s and Th2 cells (19–21). It has been suggested that the protease activity of *Alt* plays an important role in the proteolytic cleavage of IL-33_FL_ into a shorter mature form (IL-33_103-270_) (22, 23). In mice, the cleaved form of IL-33 is more potent to induce secretion of IL-5 and IL-13 by ILC2s and IL-6 and IL-13 by MC/9 mast cells than IL-33_FL_ and leads to increased eosinophilia in lungs and BALF (22). The regulation of IL-33 activity is a complex process, as *in vitro* experiments have demonstrated that the activity of the human and murine neutrophilic proteases cathepsin G and elastase may cause the generation of active mature forms of IL-33 (18, 24). Therefore, we hypothesized that neutrophils might play an important role in the regulation of IL-33 activity, which may contribute to a better understanding of the regulation of inflammation in severe airway disease. In the current paper, we have used *Alt* extract to induce airway inflammation in C57BL/6 and BALB/c mice, showing a different inflammatory response to *Alt* and observed that the induced asthma endotype is linked to different IL-33 processing *in vivo.* To study the relative contribution of neutrophils, anti-Ly6G neutralizing antibodies causing neutrophil depletion were injected into the mice, and inflammatory parameters along with cytokine profile were studied.

## Material and Methods

### Mice Experimental Procedures

All experimental procedures in mice were approved by the local Ethical Committee of Ghent University. Animals had access to food and water *ad libitum* and were kept in a 12-hour/12-hour light/dark cycle. Seven-weeks old female BALB/c or C57BL/6J wild-type mice (Janvier, Saint-Berthevin, France) were used in the study. Mice were lightly anesthetized with isoflurane/air (Ecuphar, Breda, The Netherlands) when receiving applications. For the acute model of airway inflammation, one intratracheal (i.t.) application of 20 µg of *Alt* extract (Stallergenes Greer, London, UK) in 50 µl PBS or 50 µl PBS alone (ThermoFisher Scientific, Massachusetts, USA) was given to the mice. Twelve hours after the application, the mice were euthanized with an intraperitoneal (i.p.) injection of 150 µl Dolethal (Vétoquinol, Lure, France). For the *Alt*-induced asthma model, mice were first sensitized with an i.t. application of 5 µg *Alt* extract in 50 µl PBS or 50 µl PBS and seven days later, mice were challenged every day for three times with 20 µg *Alt* extract in 50 µl PBS or 50 µl PBS as earlier described (25). Mice were euthanized twenty-four hours after the last application. For neutrophil depletion experiments, mice received an i.p. injection of 100 µg anti-Ly6G, clone 1A8 (BioXCell, New Hampshire, USA) or 100 µg isotype control antibody, clone 2A3 (BioXCell) in 200 µl PBS twenty-four hours before the 20 µg *Alt* extract (11).

### Fluorescence-Activated Cell Sorting

Murine lungs and BALF were analyzed by flow cytometry using the FACSCanto II (BD Biosciences, Erembodegem, Belgium). Murine BALF was collected by flushing the airways as described before (26) and perfused lungs were enzymatically digested using 1 mg/ml collagenase type II (Worthington Biochemical, New Jersey, USA) at 37°C for one hour. The following antibodies were used: purified CD16/CD32 (clone 93), CD11c-PE-Cy7 (clone HL3), CD11b-PerCP-Cy5.5 (clone M1/70) and Gr1-FITC (clone RB6-8C5) were purchased from ThermoFisher Scientific and Siglec F-PE (clone ES22-10D8) from Miltenyi Biotec (Bergisch Gladbach, Germany). The LIVE/DEAD Fixable Near-IR Dead Cell Stain Kit from ThermoFisher Scientific was used to exclude dead cells. The gating strategy is presented in **Supplementary Figure 2**.

### Western Blotting

Murine lungs were homogenized with the TissueLyser LT (Qiagen, Antwerp, Belgium). T-Per Tissue Protein Extraction Reagent and HALT protease inhibitor cocktail kit (ThermoFisher Scientific) were used. 25 μg total protein of lung homogenate was loaded to a 4 – 15% Mini-PROTEAN TGX Stain-Free Gels (Bio-Rad, Temse, Belgium). The proteins were separated by sodium-dodecyl sulphate-polyacrylamide gel electrophoresis and transferred to a nitrocellulose membrane (Bio-Rad). For immunostaining, the primary antibody anti-mouse-IL-33 (R&D Systems, Bio-Techne, Abingdon, UK) and secondary antibody anti-goat-horseradish peroxidase (HRP, Vector Laboratories, California, USA) were used, together with anti-β-actin (Sigma-Aldrich, Bornem, Belgium) and anti-mouse-HRP (ThermoFisher Scientific). The visualization was performed with Immobilon Western Chemiluminescence HRP substrate (Merck Millipore, Massachusetts, USA) and measured with the Chemidoc system (Bio-Rad). The band intensities were semi-quantitatively analyzed with Fiji (National Institutes of Health, Maryland, USA) by measuring the area under the peak.

### Periodic Acid-Schiff Staining

Formalin-fixed paraffin-embedded lung tissue was cut at 4 µm. The sections were stained for goblet cells using the Periodic Acid-Schiff kit (Sigma-Aldrich) conform the manufacturer’s instructions. The number of positive cells in the larger airways, with a perimeter of 600 to 2000 μm, were counted and normalized to the perimeter of airways measured using ImageJ software.

### Protein Measurement

Murine IL-4, IL-5, IL-13, IL-17, IL-25, IL-33, G-CSF and GM-CSF were measured in lung homogenates using the Mouse Magnetic Luminex assays (R&D Systems) conform the manufacturer’s protocol. Mouse TSLP and elastase were measured with the Quantikine ELISA kit (R&D Systems). Murine cathepsin G and proteinase 3 were measured with ELISA (Aviva, London, UK).

### Statistical Analysis

Data analysis was performed with Prism 9 (GraphPad, California, USA). The normality of the data was tested with a D’Agostino & Pearson test. Normally distributed data were analyzed using a one-way ANOVA with a Tukey’s test as correction for multiple comparisons, while not normally distributed data were analyzed using a one-way ANOVA Kruskal-Wallis test with a Dunn’s test as correction for multiple comparisons. Data analysis was performed with Prism 9 (GraphPad, California, USA).

## Results

### The Differential Innate Response of BALB/c Mice and C57BL/6 Mice to *Alt* Extract

**Figure 1:**
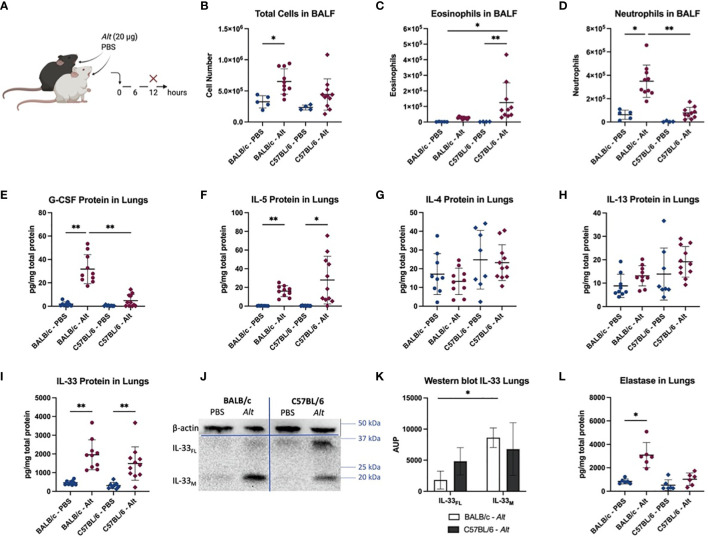
Innate response to *Alternaria alternata* (*Alt)* extract. BALB/c and C57BL/6 mice were given one intratracheal application of 20 µg *Alt* extract or PBS. Twelve hours after the application, the mice were euthanized, and samples were collected **(A)**. The total cell count **(B)** and eosinophils **(C)** and neutrophils **(D)** detected with flow cytometry in BALF are shown. The levels of G-CSF **(E)**, IL-5 **(F)**, IL-4 **(G)**, IL-13 **(H)** and IL-33 **(I)** were measured in the lung homogenates and with Luminex. Full-length IL-33 (IL-33_FL_) and cleaved, mature forms (IL-33_M_) were analyzed by western blotting and one representative blot is shown **(J)**. The area under the peak (A.U.P.) was quantified using ImageJ software **(K)**. n=4-11. The levels of elastase protein in lung homogenates is shown in **(L)**. Data is presented as mean ± SD, *P < 0.0332; **P < 0.0021.

After a single intratracheal (i.t.) application of *Alt* extract (**Figure 1A**), BALB/c mice showed significantly higher numbers of total BALF cells compared to PBS-treatment (**Figure 1B**). The BALF of C57BL/6 mice mostly contained eosinophils (**Figure 1C**), while BALB/c mice responded with a neutrophilia (**Figure 1D**). As expected, the neutrophilic inflammation in BALB/c was accompanied by an upregulation of G-CSF (**Figure 1E**) in the lungs 12 hours after the *Alt* treatment, consistent with the function of G-CSF in promoting the recruitment of neutrophils from the bone marrow after airway allergen challenge (27, 28). Remarkably, both mouse strains showed a fast upregulation of IL-5 in the lungs after the *Alt* treatment (**Figure 1F**). However, there was a trend towards higher production of IL-5 in the lungs of C57BL/6 mice in response to *Alt*. The levels of lung IL-4, IL-13, IL-17, IL-25, GM-CSF were either not detectable or not different from the PBS group in both mouse strains (**Figures 1G, H** or data not shown). IL-33 protein also showed a fast upregulation in both mouse strains after treatment with *Alt*, as IL-5 (**Figure 1I**). In BALB/c mice, the cleaved mature form of IL-33 (IL-33_M_; ~ 18 kDa) was significantly more pronounced in the lungs, while in C57BL/6 mice also full-length IL-33 (IL-33_FL_; ~ 30 kDa) was present (**Figures 1J, K**). The different processing of IL-33 between the strains was suggestive for a role of neutrophilic proteases in cleavage of IL-33_FL_ in BALB/c mice, as the elastase levels in the lungs of BALB/c showed a significant increase when treated with *Alt* extract (**Figure 1L**). The neutrophilic proteases cathepsin G and proteinase 3 could not be measured with the used ELISA kits (data not shown).

### Differential Immune Response in C57BL/6 and BALB/c Mice Persists After Multiple Sensitizations With *Alt* Extract

**Figure 2:**
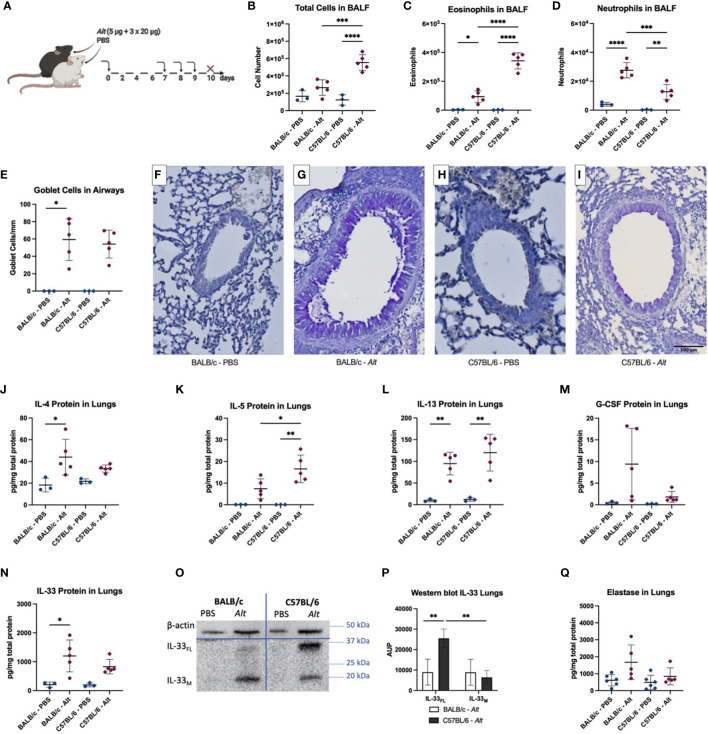
BALB/c and C57BL/6 mice were sensitized with one intratracheal (i.t.) application of 5 µg *Alternaria alternata* (*Alt)* extract or PBS. On days 7, 8 and 9 the mice were challenged i.t. with 20 µg *Alt* extract or PBS. Twenty-four hours after the application mice were euthanized, and samples were collected **(A)**. The total cell count **(B)**, eosinophils **(C)** and neutrophils **(D)** in BALF determined by flow cytometry. Quantification of goblet cells in the larger airways **(E)** and representative images of PAS-staining for the groups BALB/c – PBS **(F)**, BALB/c – *Alt* **(G)**, C57BL/6 – PBS **(H)** and C57BL/6 – *Alt* **(I)**. The levels of IL-4 **(J)**, IL-5 **(K)**, IL-13 **(L)**, G-CSF **(M)** and IL-33 **(N)** were analyzed in lung homogenates by Luminex. Full-length IL-33 (IL-33_FL_) and mature forms (IL-33_M_) were analyzed by western blotting and area under the peak (A.U.P.) was quantified using ImageJ software **(P)**. One representative blot is shown **(O)**. Elastase protein levels in the lungs are shown in **(Q)**. n=3-5. Data is presented as mean ± SD, *P < 0.0332; **P < 0.0021; ***P < 0,0002; ****P < 0,0001.

To test if the mice would keep the differential inflammatory pattern after multiple applications, mice were sensitized i.t. with 5 µg of *Alt* extract and then challenged with 20 µg of *Alt* extract i.t. on days 7, 8, and 9 (**Figure 2A**). In contrast to a single *Alt* application, the total BALF count was especially upregulated in C57BL/6 mice treated with *Alt* (**Figure 2B**). The numbers of eosinophils (**Figure 2C**) and neutrophils (**Figure 2D**) were upregulated in both mouse strains after *Alt* treatment. However, eosinophils were significantly higher in case of C57BL/6 mice, while the BALF of BALB/c mice had significantly higher numbers of neutrophils after the *Alt* treatment. Goblet cells were significantly increased in BALB/c mice and showed a trend for increase in C57BL/6 mice after *Alt* treatment compared to PBS (**Figures 2E–I**). IL-4 was significantly increased in the lungs of BALB/c mice, while IL-5 showed a significant upregulation in the lungs of C57BL/6 mice after *Alt* treatment (**Figures 2J, K**). The cytokine IL-13 was upregulated in the lungs of both mouse strains after *Alt* treatment (**Figure 2L**). G-CSF protein levels in the lungs were not different between the groups, however, a trend for upregulation in BALB/c mice after *Alt* could be observed (**Figure 2M**). The levels of GM-CSF, IL-17, and TSLP were below detection level in the lungs of mice exposed to PBS or *Alt* extract, while the levels of IL-25 were not different between the groups (data not shown). IL-33 protein levels were higher in the lungs of BALB/c mice after *Alt* treatment, and a trend for an increase in C57BL/6 could be observed (**Figure 2N**). On western blot, IL-33_FL_ was more abundant in the lungs of C57BL/6 mice compared to BALB/c mice (**Figures 2O, P**). The levels of neutrophilic elastase show a trend for an increase in BALB/c treated with *Alt* extract compared to PBS treatment, but not in C57BL/6 mice (**Figure 2Q**).

### Neutrophils Could Control the Immune Response to *Alternaria via* IL-33 Cleavage in BALB/c Mice

**Figure 3:**
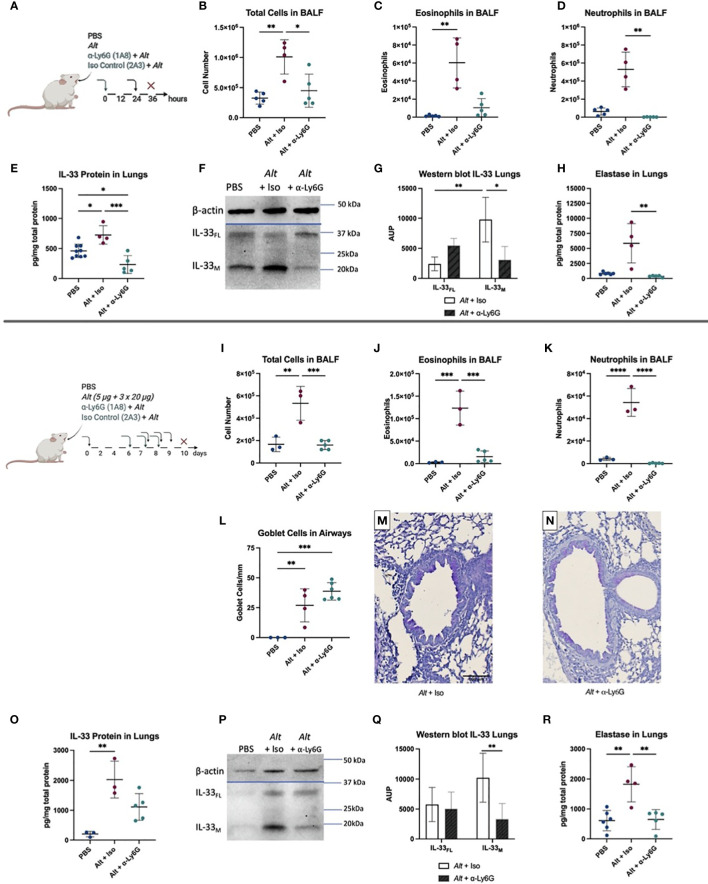
BALB/c were given one i.p. injection of 100 µg anti-Ly6G antibody or IgG2a isotype control (Iso). After 24 hours, they received one i.t. application of 20 µg *Alternaria alternata* (*Alt)* extract or PBS. Twelve hours later the mice were euthanized, and samples were collected **(A)**. The total cell count **(B)**, eosinophils **(C)**, neutrophils **(D)** in BALF determined by flow cytometry. IL-33 protein levels **(E)** and one representative western blot to detect full-length IL-33 (IL-33_FL_) and cleaved forms (IL-33_M_) **(F)** with quantification of area under the peak (A.U.P.) **(G)** are shown. Elastase protein levels, measured by Luminex, in the lung are presented **(H)**. Next, BALB/c mice were first sensitized with one i.t. application of 5 µg *Alt* extract or PBS. On days 7, 8 and 9 the mice got challenged by i.t. applications of 20 µg *Alt* extract or PBS. Twenty-four hours before each i.t., an i.p. of 100 µg anti-Ly6G antibody clone 1A8 or IgG2a isotype control clone 2A3 was given. Twenty-four hours after the last application mice were euthanized, and samples collected: the total cell count **(I)**, eosinophils **(J)** and neutrophils **(K)** in BALF were analyzed by flow cytometry. Quantification of goblet cells in the larger airways **(L)** and representative images of PAS staining for the groups *Alt* + Iso **(M)** and *Alt* + anti-Ly6G **(N)**. The levels of IL-33 **(O)** in the lung homogenates were analyzed by Luminex. One representative blot for IL-33 western blotting **(P)** in the lungs is shown with quantification of A.U.P. **(Q)**. Finally, the level of elastase in lung homogenates, determined by Luminex **(R)**. n=3-9. Data is presented as mean ± SD, *P < 0.0332; **P < 0.0021; ***P < 0,0002; ****P < 0,0001.

Since BALB/c mice showed a more prominent neutrophilic response, we investigated the regulatory role of neutrophils in the innate phase of *Alt*-induced asthma. BALB/c mice were injected intraperitoneally (i.p.) with anti-Ly6G neutralizing antibodies or an isotype control antibodies prior to a single i.t. application of *Alt* extract (**Figure 3A**). The injection of neutralizing antibody lead to a decrease in the total BALF cell number and a trend for a decrease of eosinophils in the BALF (**Figures 3B, C**). It resulted, as expected, in the reduced numbers of neutrophils in the BALF (**Figure 3D**). Neutrophil depletion did not affect the levels of cytokines with the exception of IL-33 (**Figures 3E** and **Supplementary Figures 1A–D**). The western blot indicated a significant decrease of mature IL-33 (**Figures 3F, G**) after neutrophil depletion. As expected, a significant drop in neutrophil numbers in the lungs of anti-Ly6G injected BALB/c mice induced a strong significant reduction of neutrophilic elastase after one application of *Alt* (**Figure 3H**).

To test the role of neutrophil depletion in the sensitization and challenge stage of allergic inflammation, mice were treated with *Alt* extract as described above. Twenty-four hours before each challenge with *Alt* mice were injected with anti-Ly6G antibody (**Figures 3I–R**). The total cell number and the number of eosinophils was significantly reduced in BALF of mice treated with anti-Ly6G antibody (**Figures 3I, J**). We do not exclude that about 10% of the depleted eosinophils express Ly6G^+^ and therefore could also be affected (29). The depletion of neutrophils was complete in the BALF of BALB/c mice treated with anti-Ly6G antibody (**Figure 3K**). The levels of G-CSF, IL-4, IL-5, and IL-13 cytokines in the lungs were not different to the isotype control group (**Supplementary Figures 1E–H**), as were the elevated numbers of mucus producing Goblet cells, that were not affected by anti-Ly6G treatment (**Figures 3L–N**). A trend to reduced total IL-33 levels in the lungs was seen after neutrophil depletion, however, it didn’t reach significance (**Figure 3O**). Importantly, western blot has demonstrated a significant reduction of the cleaved form of IL-33 in mice exposed to *Alt* in the absence of neutrophils compared to controls, consistent with the reduced number of inflammatory cells in the airways of mice injected with anti-Ly6G antibody (**Figures 3P, Q**). Neutrophil depletion led as expected to a significant decrease of neutrophilic elastase in the long-term protocol (**Figure 3R**). These observations suggest a role for neutrophilic proteases in the regulation of IL-33 in type 2 immune responses to *Alt.*

## Discussion

Activation of airway epithelium with allergens including *Alt* extract often causes severe asthma attacks and results in the release of several alarmins including IL-33, IL-25, GM-CSF, TSLP which orchestrate the downstream type 2 immune response (30, 31). It has been thought that in mice, *Alt* induces exclusively type 2 biased immune responses by initiating the release of IL-33 and activating ILC2s and Th2-cells in the airways (11, 30). Due to sensing of the allergen through the proteinase-activated receptor 2 receptor, the cells release ATP, which leads to an increase in intracellular Ca^2+^ concentration and the subsequent rapid active release of IL-33 (30, 32). Our study has shown that C57BL/6 and BALB/c mice have significant amounts of IL-33 in the lungs after i.t. application of *Alt* extract. However, we demonstrated a different cleavage pattern of IL-33_FL_. Namely, in C57BL/6 mice both IL-33_FL_ and IL-33_M_ were upregulated. Interestingly, *Alt* exposure in BALB/c lead to the cleavage of IL-33_FL_ resulting in the exclusive presence of IL-33_M_ in the lungs. Likewise, mice sensitized and challenged with *Alt* extract had a similar IL-33 cleavage patterns with significantly lower levels of IL-33_FL_ in the lungs of BALB/c mice, associated with neutrophilic infiltration. In contrast to the paper by Cayrol et al. (22) showing that IL-33 could be proteolytically cleaved by allergens, including *Alt*, our study stresses the role of the endogenous regulation of IL-33 activity (33). The proteolytic cleavage of IL-33 is an important regulatory mechanism in allergic airway inflammation and a balance between protease and protease inhibitors is crucial for this regulation. Moreover, endogenous serine protease inhibitors could play a role in the regulation of IL-33 activity by acting on endogenous proteases (34). Cellular proteases from mast cells and neutrophils were shown to change the activity of murine IL-33 and influence the outcome of the inflammatory response (19, 24). Activated neutrophils release the proteases cathepsin G, elastase and proteinase 3 simultaneously into the extracellular space (18). The activity of IL-33 was shown to be robustly increased when full-length murine IL-33_1-266_ gets cleaved *in vitro* by elastase or cathepsin G, generating the shorter forms IL-33_102-266_ and IL-33_109-266_, while proteinase-3 activates or inactivates the cytokine *in vitro*, dependent on incubation times and the concentrations of other neutrophilic proteases present (18, 24, 35). We have shown that neutrophils could contribute to the regulation of IL-33 processing *in vivo* in an *Alt*-induced asthma model. Depletion of neutrophils *in vivo* by an *i.p.* injection of anti-Ly6G antibodies in BALB/c mice resulted in the significant decrease of elastase, next to the significant reduction of IL-33_M_ after i.t. *Alt* applications. This suggests the role of neutrophilic proteases in the regulation of IL- 33 activity. Remarkably, the IL-4, IL-5 and IL-13 levels were not affected by neutrophil depletion, possibly indicating the role of ILC2 in the *Alt* exposed mice as described earlier (36). The presence of the mucus-producing cells was as a result also not affected. Several studies have demonstrated that IL-33 is a key cytokine induced by allergens triggering eosinophilic inflammation (7, 25, 26). Our current study suggests that neutrophils could control allergic immune responses at its innate and adaptive stage and contribute to the processing of IL-33_FL_. In contrast, the study by Patel et al. has shown that neutrophils restrain allergic airway inflammation by limiting ILC2 function and dendritic cell antigen presentation in HDM-induced asthma in BALB/c mice (11).

We have demonstrated that *Alt* extract elicits two distinct inflammatory endotypes at innate immune response stages in BALB/c and C57BL/6 mice. Twelve hours after *Alt* exposure, C57BL/6 mice showed an eosinophilic type 2 biased airway inflammation. On the contrary, BALB/c mice showed a neutrophilic influx in the BALF, increased G-CSF levels and a specific IL-33 cleavage pattern in the lungs. BALB/c mice already showed increased G-CSF levels in the lungs at six hours after a single application with *Alternaria*, while IL-33 and IL-5 levels were not different between the strains (data not shown). Remarkably, this initial innate response persisted after multiple *Alt* challenges. During sensitization and challenge phases, C57BL/6 mice showed increased levels of IL-5 and a stronger eosinophilic inflammation, while BALB/c mice did not. On the contrary to C57BL/6 mice, BALB/c mice showed higher levels of neutrophils.

The strain specific difference in mice has been noted before. The ovalbumin treated C57BL/6J mice have a massive influx of eosinophils and neutrophils in the airways, while BALB/c only showed a modest to weak response (37). At the same time airway responsiveness was more increased in BALB/c mice compared to C57BL/6J (37, 38). House dust mite treated C57BL/6J mice showed a more robust inflammatory response, higher airway eosinophilia, type 2 cytokines and IgE levels than BALB/cJ mice, but a lower airway responsiveness (39). This is not surprising since the genetic analysis using a mouse tool has demonstrated that these strains are different in more than 7000 unique mutations (40). Our study shows the importance of the choice of mouse strains as outcomes, such as IL-33 and neutrophil/eosinophil influx, can be different after the challenge with the same allergen. It also suggests that early innate immune response might dictate an immunological bias at the later stages of airway disease. The differential cleavage pattern in C57/BL6J and BALB/c mice could be explained by differential expression of endogenous serine protease inhibitors (serpins), which play a crucial play a role in the regulation of IL-33 activity (33, 34).

In conclusion, in the current study we extend our knowledge on the regulatory mechanisms of IL-33 in the airway inflammation. Our study demonstrates that different inbred mouse strains become a useful tool for studying the endotypes of inflammatory airway diseases with BALB/c mice showing a mixed inflammatory response characterized by neutrophilic inflammation and typical type 2 cytokine profile. The use of different mouse strains is a largely underestimated tool that could offer more insights in understanding the complex interactions of immune cells occurring during chronic airway inflammation in human patients. Neutrophils play a crucial role in control of allergic type 2 immune response towards *Alt* extract and affect processing of IL-33_FL_ in the lungs.

## Data Availability Statement

The raw data supporting the conclusions of this article will be made available by the authors, without undue reservation.

## Ethics Statement

The animal study was reviewed and approved by Ethical Committee of Ghent University.

## Author Contributions

OK designed and performed experiments, analyzed data and wrote the original draft. SN performed experiments, analyzed data, wrote the manuscript. JO, GH, NZ, HB, and NR performed experiments and analyzed the data. TM and HB provided analysis tools and revised the manuscript. CB supervised the study, provided analysis tools, and revised the manuscript. All authors contributed to the article and approved the submitted version.

## Funding

The study has been supported by FWO grant 3G065319 to CB and OK, and FWO grant 3G041819 to TM. SN is a doctoral fellow paid by the Fund for Scientific Research Flanders - Strategic Basic Research (3S035019).

## Conflict of Interest

The authors declare that the research was conducted in the absence of any commercial or financial relationships that could be construed as a potential conflict of interest.

## Publisher’s Note

All claims expressed in this article are solely those of the authors and do not necessarily represent those of their affiliated organizations, or those of the publisher, the editors and the reviewers. Any product that may be evaluated in this article, or claim that may be made by its manufacturer, is not guaranteed or endorsed by the publisher.

## Supplementary Material

Supplementary Figure 1 BALB/c were given one i.p. injection of 100 µg anti-Ly6G antibody or IgG2a isotype control (Iso). After 24 hours, they received one i.t. application of 20 µg *Alternaria alternata* (*Alt)* extract or PBS. Twelve hours later the mice were euthanized. Levels of G-CSF **(A)**, IL-4 **(B)**, IL-5 **(C)** and IL-13 **(D)** were analyzed *via* Luminex measurements in lung homogenates. Next, BALB/c mice were first sensitized with one i.t. application of 5 µg *Alt* extract or PBS. On days 7, 8 and 9 the mice got challenged by i.t. applications of 20 µg *Alt* extract or PBS. Twenty-four hours before each i.t., an i.p. of 100 µg anti-Ly6G antibody clone 1A8 or IgG2a isotype control clone 2A3 was given. Twenty-four hours after the last application mice were euthanized, and samples collected. Levels of G-CSF **(E)**, IL-4 **(F)**, IL-5 **(G)** and IL-13 **(H)** were analyzed in lung homogenates with Luminex. n=3-9. Data is presented as mean ± SD, *P < 0.0332; **P < 0.0021; ***P < 0,0002.

Supplementary Figure 2 Gating strategy used for flow cytometry to analyze of eosinophils and neutrophils. First, the subset of viable cells is gated through live/dead staining. Next, cells are gated for the CD11c^-^ population. From this subset, eosinophils are defined as CD11b^+^, SiglecF^+^ and neutrophils as SSC^low^, CD11b^+^, Gr1^+^.
