# Supplementary figures and images for: Neutrophils Affect IL-33 Processing in Response to the Respiratory Allergen Alternaria alternata
Source: Front Immunol. 2021 Aug 17;12:677848. doi: 10.3389/fimmu.2021.677848 (PMC8416032; doi:10.3389/fimmu.2021.677848)

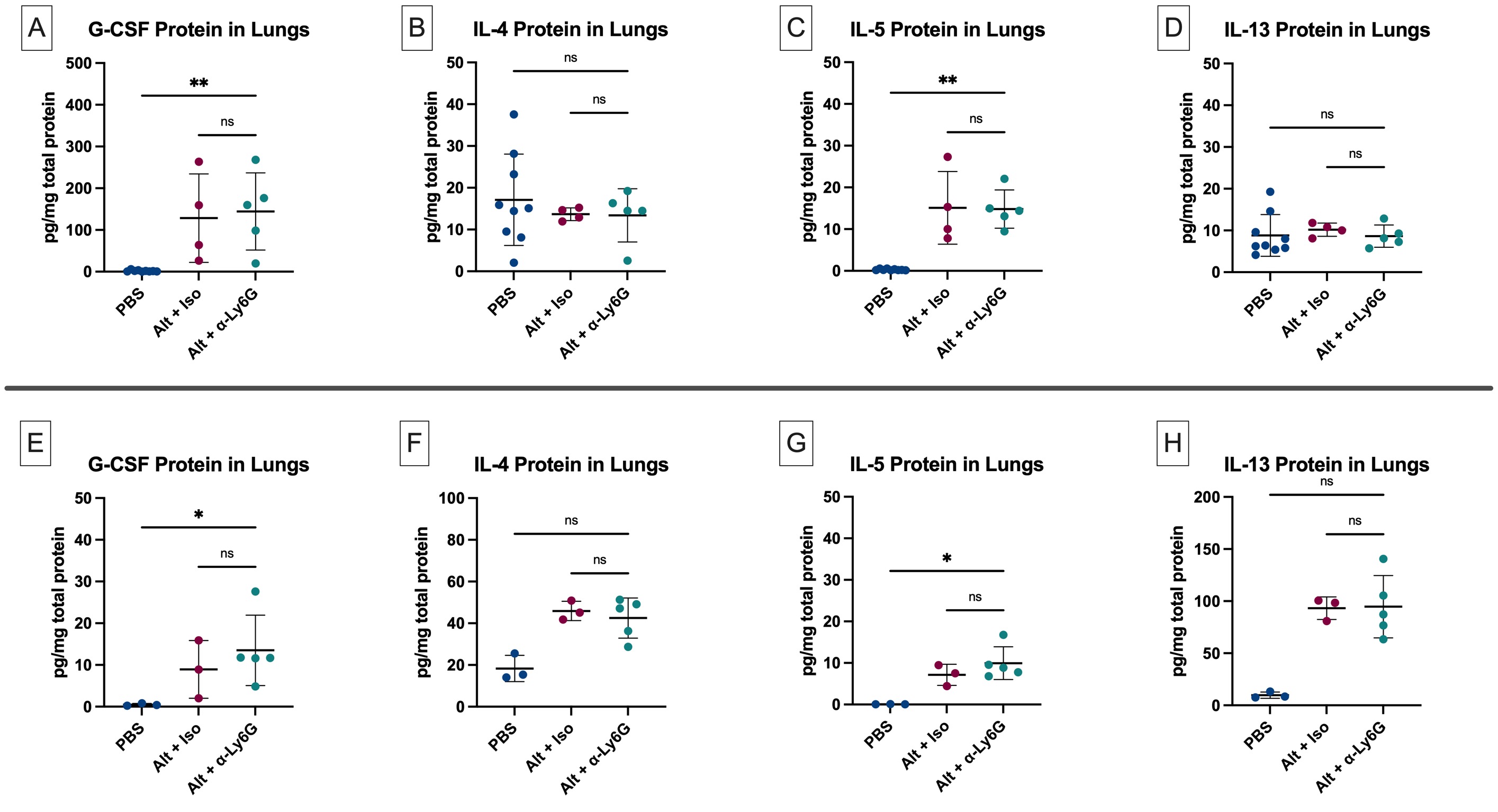

Supplement: Supplementary Figure 1 — BALB/c were given one i.p. injection of 100 µg anti-Ly6G antibody or IgG2a isotype control (Iso). After 24 hours, they received one i.t. application of 20 µg Alternaria alternata (Alt) extract or PBS. Twelve hours later the mice were euthanized. Levels of G-CSF (A), IL-4 (B), IL-5 (C) and IL-13 (D) were analyzed via Luminex measurements in lung homogenates. Next, BALB/c mice were first sensitized with one i.t. application of 5 µg Alt extract or PBS. On days 7, 8 and 9 the mice got challenged by i.t. applications of 20 µg Alt extract or PBS. Twenty-four hours before each i.t., an i.p. of 100 µg anti-Ly6G antibody clone 1A8 or IgG2a isotype control clone 2A3 was given. Twenty-four hours after the last application mice were euthanized, and samples collected. Levels of G-CSF (E), IL-4 (F), IL-5 (G) and IL-13 (H) were analyzed in lung homogenates with Luminex. n=3-9. Data is presented as mean ± SD, *P < 0.0332; **P < 0.0021; ***P < 0,0002. [file Image_1.jpeg]

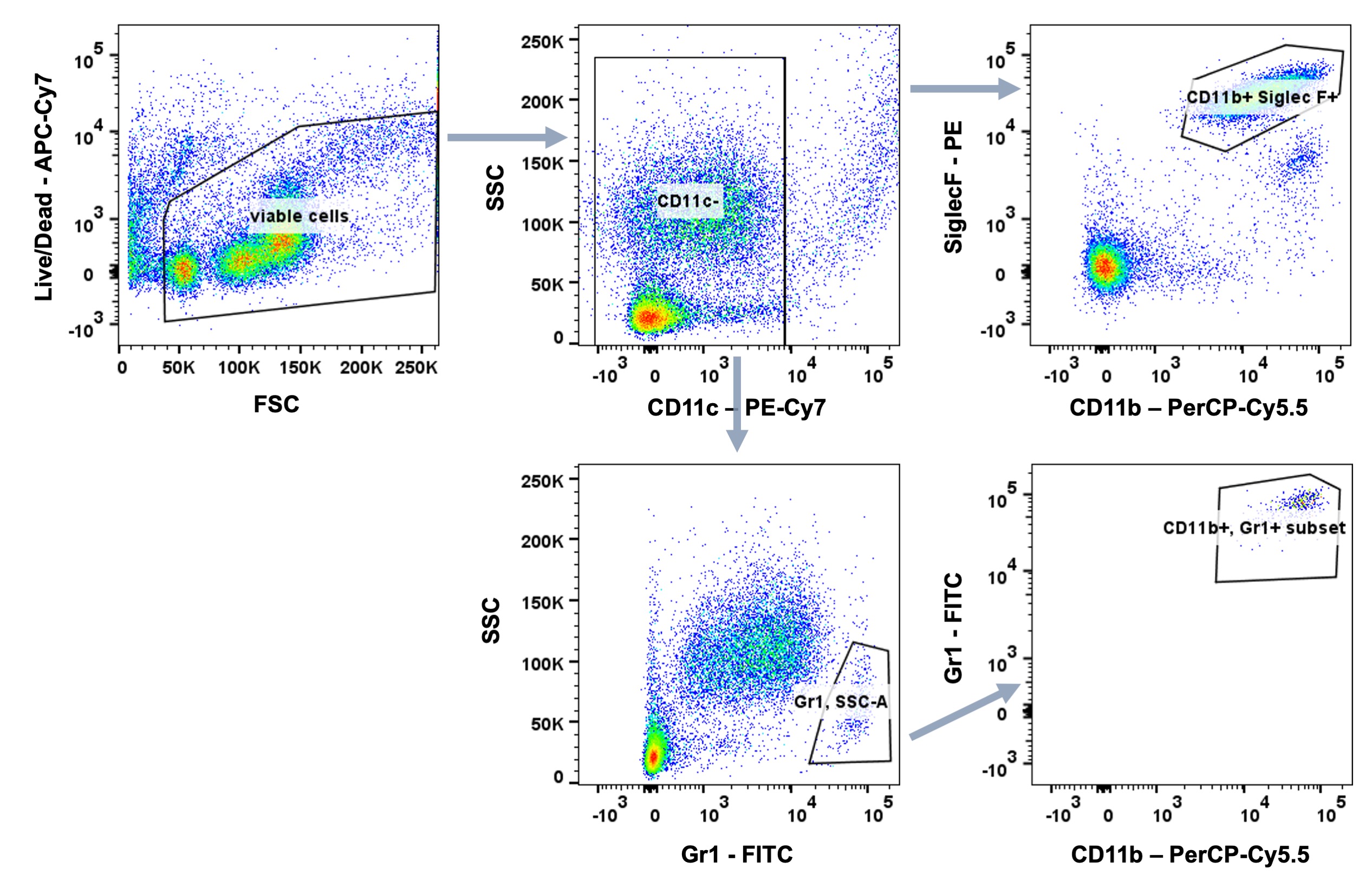

Supplement: Supplementary Figure 2 — Gating strategy used for flow cytometry to analyze of eosinophils and neutrophils. First, the subset of viable cells is gated through live/dead staining. Next, cells are gated for the CD11c- population. From this subset, eosinophils are defined as CD11b+, SiglecF+ and neutrophils as SSClow, CD11b+, Gr1+. [file Image_2.jpeg]
